# Supplementary material for: Spin polarized Fe1−Ti pairs for highly efficient electroreduction nitrate to ammonia
Source: Nat Commun. 2024 Jan 2;15:88. doi: 10.1038/s41467-023-44469-4 (PMC10762114; doi:10.1038/s41467-023-44469-4)
Supplement: Supplementary file 1 — Supplementary Information [file 41467_2023_44469_MOESM1_ESM.pdf]

Supplementary Information

Spin polarized Fe_1–Ti pairs for highly efficient electroreduction nitrate to ammonia

Jie Dai[1‡], Yawen Tong[2‡], Long Zhao[1‡], Zhiwei Hu[3], Chien-Te Chen[4], Chang-Yang Kuo[4,5], Guangming Zhan[1], Jiaxian Wang[1], Xingyue Zou[1], Qian Zheng[1], Wei Hou[1], Ruizhao Wang[1], Kaiyuan Wang[1], Rui Zhao[1], Xiang-Kui Gu[2]*, Yancai Yao[1]* and Lizhi Zhang[1]*

[1]School of Environmental Science and Engineering, Shanghai Jiao Tong University, Shanghai 200240, China.

[2]School of Power and Mechanical Engineering, Wuhan University, Wuhan 430072, China.

[3]Max Planck Institute for Chemical Physics of Solids, Nothnitzer Strasse 40, 01187 Dresden, Germany.

[4]National Synchrotron Radiation Research Center, 101 Hsin-Ann Road, Hsinchu 30076, Taiwan, R.O.C.

[5]Department of Electrophysics, National Yang Ming Chiao Tung University, Hsinchu, Taiwan, R.O.C.

[‡]These authors contributed equally to this work.

*Correspondence to: xiangkuigu@whu.edu.cn; yyancai@sjtu.edu.cn; zhanglz@ccnu.edu.cn

Experimental details

Computational methods. Spin–polarized DFT calculations were performed by the Vienna ab initio simulation package (VASP) [2, 3]. The exchange–correlation interaction was described by the PBE functional[4]. The Kohn–Sham equations were solved in a plane wave basis set with a kinetic energy cutoff of 400 eV. The effect of van der Waals interaction was described using the dispersion–corrected DFT–D3 functional[5]. Ab–initio molecular dynamics (AIMD) simulations were used to generate the amorphous structure of TiO_2 through the melt–and–quench method, which has been successfully utilized to generate the atomistic models of amorphous oxides[6–8]. The rutile TiO_2 containing 96 atoms underwent molecular dynamics for a duration of 10 ps at a temperature of 2500 K (higher than the melting point 2116 K of TiO_2 crystal)[9]. We further cooled the model to 300 K at the rate of 110 K/ps to obtain the amorphous structure of TiO_2. To model SD–Fe_1–Ti, various possible sites of single Fe atom deposited on surface were considered and the most stable structure was displayed in Fig. 5b, where he Fe atom was stabilized by four Fe–O bonds, consistent well with our EXAFS result (Supplementary Table 3). To model SP–Fe_1–Ti, we considered a single Fe atom anchored an OV on the surface according to the ESR result. It was found that the signal intensity at g = 2.008, assigned to OVs, was decreased after the introduction of Fe metal ions on TiO_x, indicating Fe atoms may be anchored on OVs[1, 10]. Optimized structure displayed that Fe atom coordinated with three O atoms forming three–coordinate configuration, in line well with the EXAFS result. A vacuum space along the z direction was set to 12 Å to prevent the interaction between the repeating slabs. A k–point mesh of (3 × 3 × 1) was used to sample the slab Brillouin zone. Structure relaxation was performed until the residual forces were less than 0.02 eV $Å^{-1}$. To correct the strong electron–correlation properties of transition metal oxides, DFT+U calculations[11] were performed with U_{eff} Vaules of 3.5 and 2 eV for Ti–3d and Fe–3d, based on the literature[12]. The crystal orbital Hamilton population (COHP) was analyzed by the LOBSTER program[13, 14].

The adsorption energies (E_{ads}) of the adsorbed species were described by the following equation:

$$E_{ads} = E_{total} - E_{adsorbate} - E_{slab}$$

where E_{total}, $E_{adsorbate}$ and $E_{surface}$ are the total energies of the adsorbate–slab complex, the adsorbate and slab, respectively. The Gibbs free energy change (ΔG) of the elementary step was estimated by

$$\Delta G = \Delta E_{DFT} + \Delta E_{ZPE} - T\Delta S$$

where ΔE_{DFT} is the change in the electronic energy difference calculated by DFT, ΔE_{ZPE} is the change in the zero–point energy, T is the room temperature (300 K) and ΔS is the entropy change. To avoid using the charged NO_3^- species as a reference, the neutral HNO_3 was alternatively used to calculate the Gibbs free energy of NO_3 on the basis of literature[15–17], and the $\Delta G(*NO_3)$ can be calculated as

$$\Delta G_{*NO_3} = G_{*NO_3} - G_{*} - G_{HNO_3(g)} + 0.5G_{H_2(g)} + \Delta G_{correct}$$

$$\Delta G_{correct} = \Delta G_{S1} + \Delta G_{S2}$$

where G_{*NO_3}, G_{*}, $G_{HNO_3(g)}$ and $G_{H_2(g)}$ are the Gibbs free energies of adsorbed NO_3, clean substrate, HNO_3 and H_2 molecules in the gas phase, respectively. $\Delta G_{correct}$ denotes the correction of adsorption energy. ΔG_{S1} and ΔG_{S2} are the Gibbs free energy of formation of HNO_3(l) from NO_3^-(aq) (0.317 eV) and the Gibbs free energy of vaporization of HNO_3(l) (0.075 eV). Both values can be obtained from the CRC handbook, and the same approach has been done in literature[15–17].

Supplementary Figures

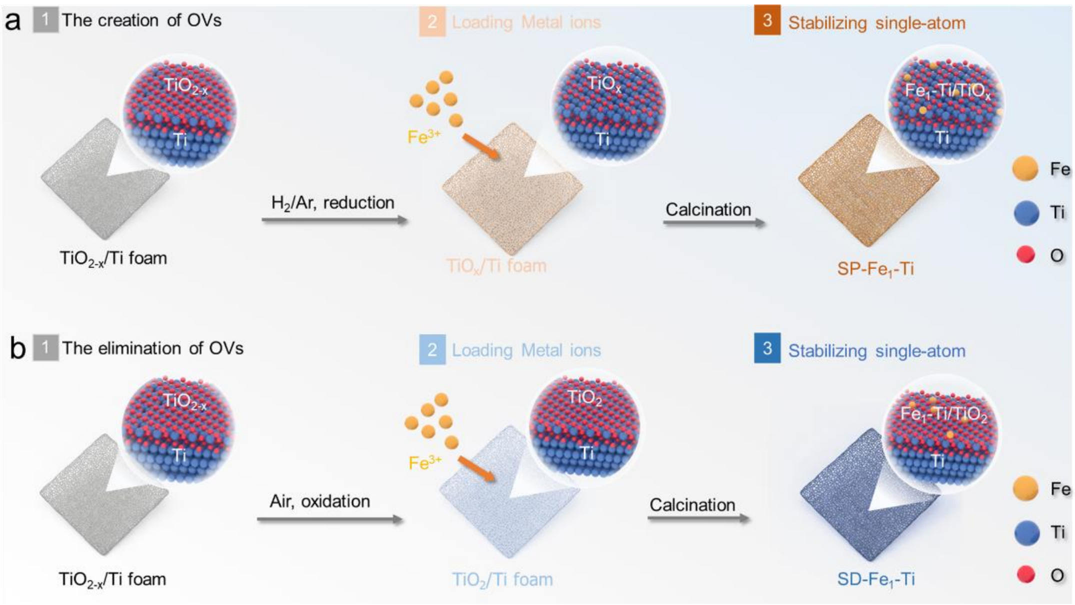

Supplementary Figure 1. The typical synthesis route of SP–Fe_1–Ti and SD–Fe_1–Ti electrodes. The schematical illustration for the preparation of (a) SP–Fe_1–Ti and (b) SD–Fe_1–Ti electrodes. Color code: Ti (blue), Fe (yellow) and O (red).

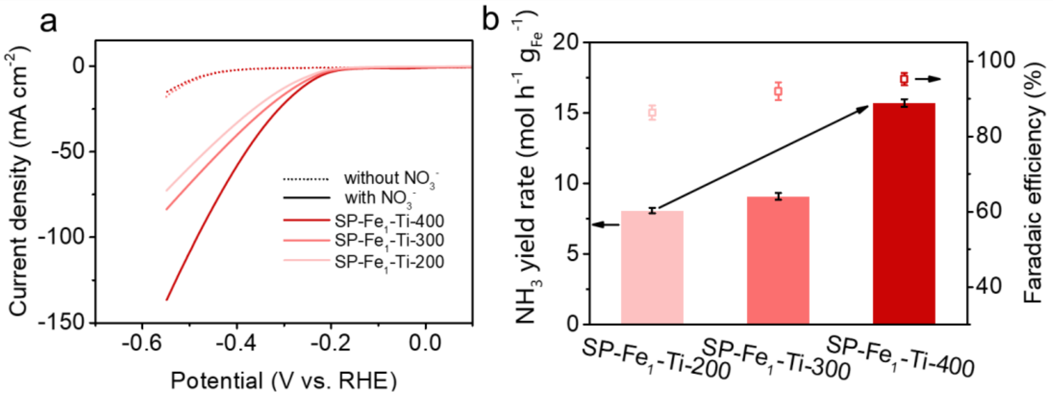

Supplementary Figure 2. NITRR activity of SP−Fe_1−Ti electrode annealing at different temperatures. (a) LSV curves of SP−Fe_1−Ti electrode annealing at different temperatures (200, 300, 400 °C) in 1 mol L^{-1} KOH with or without $NaNO_3$. (b) NH_3 yield rate and FE_{NH3} of SP−Fe_1−Ti electrode annealing at different temperatures (200, 300, 400 °C) at –0.4 V vs. RHE. The error bars correspond to the standard deviation from three independent measurements. We also prepared the SP−Fe_1−Ti electrode at 500 °C, but it cannot keep the mechanical stability and cracked, which may result from the hydrogen embrittlement.

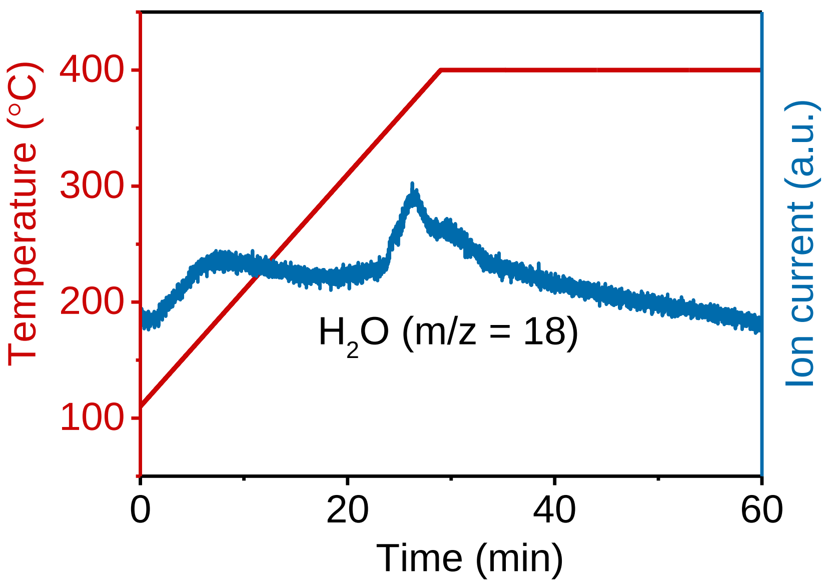

Supplementary Figure 3. Investigation of the formation mechanism for oxygen vacancies. Mass spectroscopy during the temperature programmed reaction (TPR) measurement of pristine Ti foam in 5% H_2/Ar.

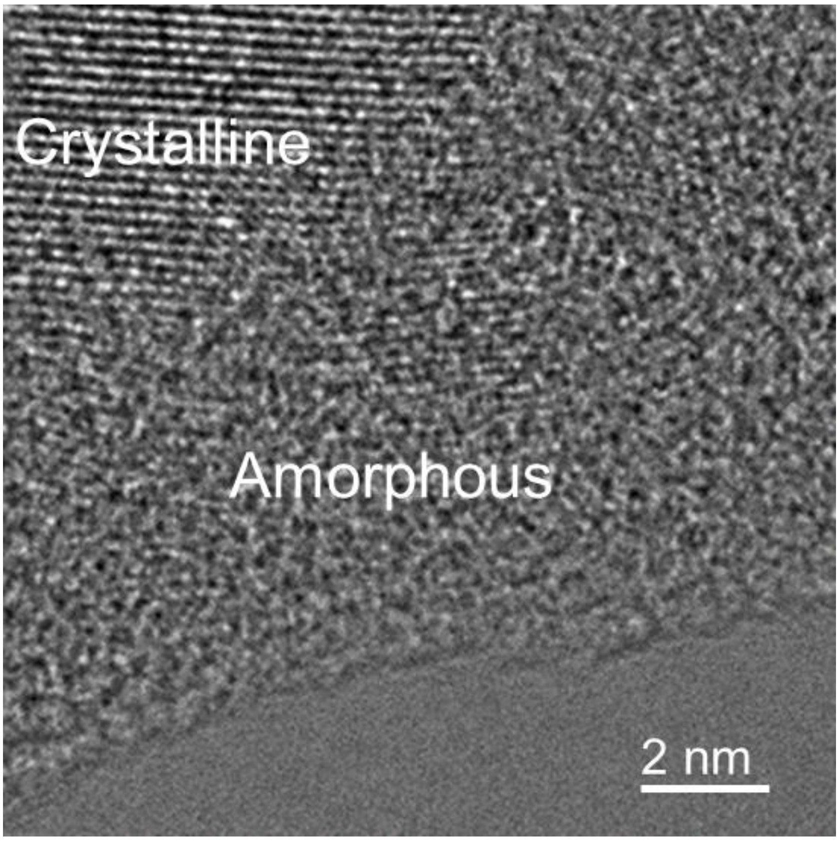

Supplementary Figure 4. HRTEM image of SP–Fe_1–Ti electrode. As visualized by the HRTEM image of SP−Fe_1−Ti electrode, a distinct boundary was observed between the interior crystalline Ti and surface amorphous TiO_x layer, consistent with our previous work[1].

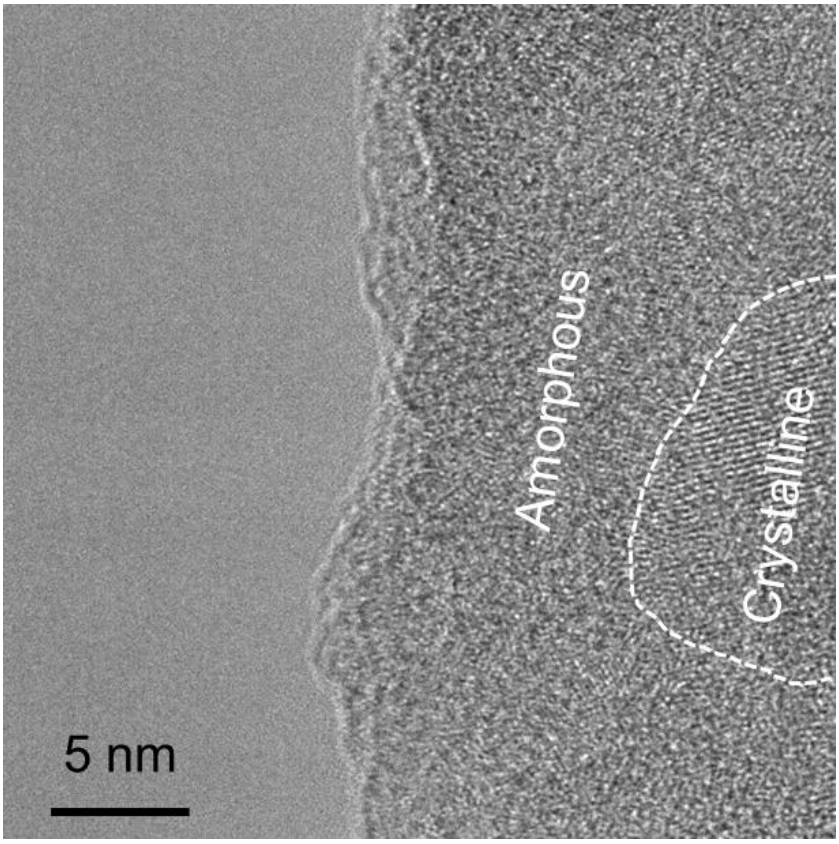

Supplementary Figure 5. HRTEM image of SD–Fe_1–Ti electrode. As visualized by the HRTEM image of SD−Fe_1−Ti electrode, a distinct boundary was observed between the interior crystalline Ti and surface amorphous TiO_x layer, which was similar with SP−Fe_1−Ti.

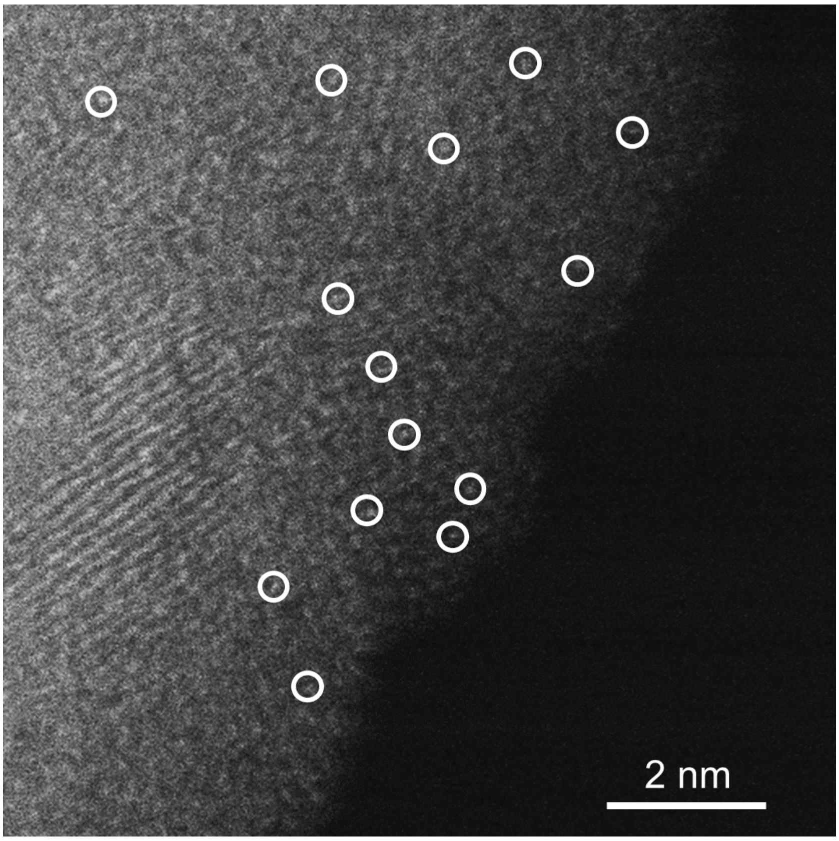

Supplementary Figure 6. HAADF–STEM image of SD−Fe_1−Ti electrode. The HAADF−STEM image demonstrated that single–atom Fe (marked by white circles) was well dispersed on the oxide layer of Ti foam for SD–Fe_1–Ti electrode.

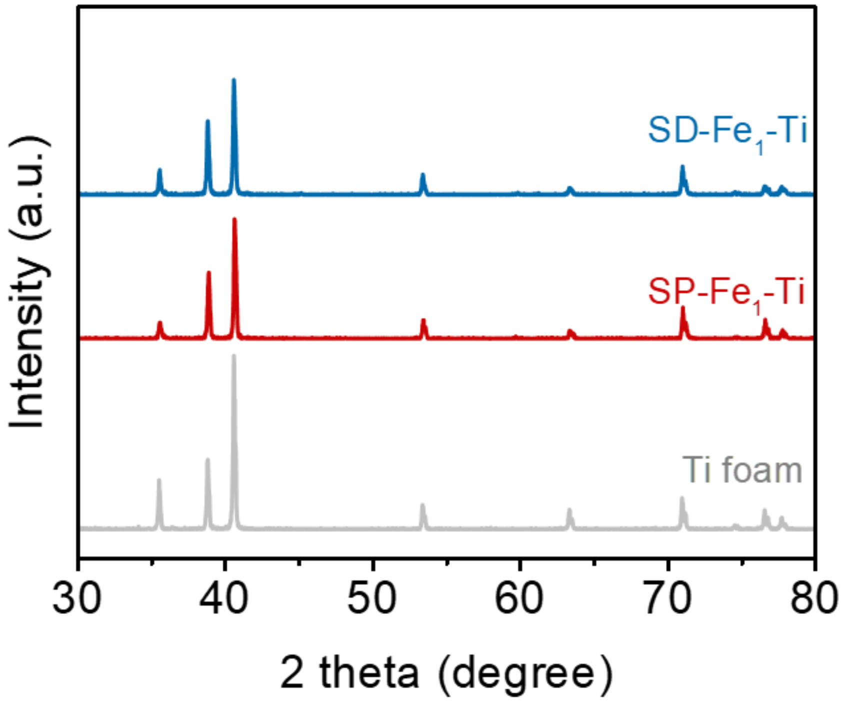

Supplementary Figure 7. XRD patterns of Ti foam, SD–Fe_1–Ti and SP–Fe_1–Ti electrodes. The X−ray diffraction (XRD) patterns of SD–Fe_1–Ti and SP–Fe_1–Ti electrodes excluded diffraction peaks of the metallic Fe crystalline.

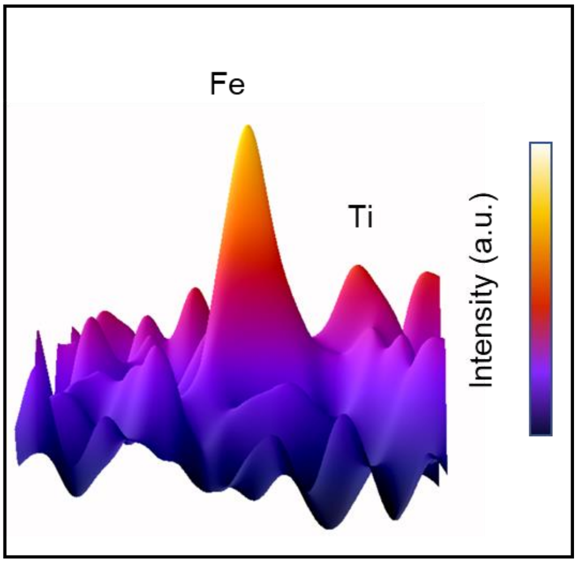

Supplementary Figure 8. 3D surface intensity profile. Intensity profiles were taken along the yellow arrow in HAADF–STEM image. The 3D surface intensity profile along the yellow arrow and the corresponding showed the characteristics of single−site Fe on the Ti monolithic electrode.

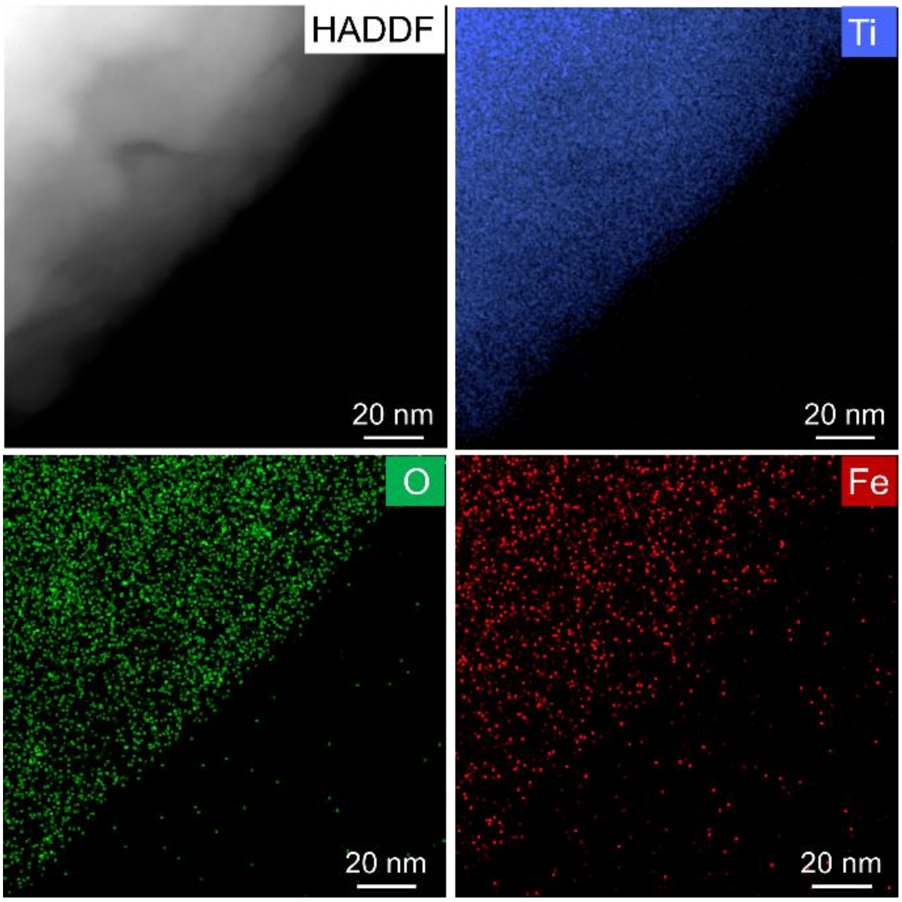

Supplementary Figure 9. HADDF image and STEM elemental mapping of SD–Fe$_1$–Ti electrode. The EDS elemental mapping images revealed the uniform dispersion of Fe atoms on the surface of SD−Fe$_1$−Ti electrode.

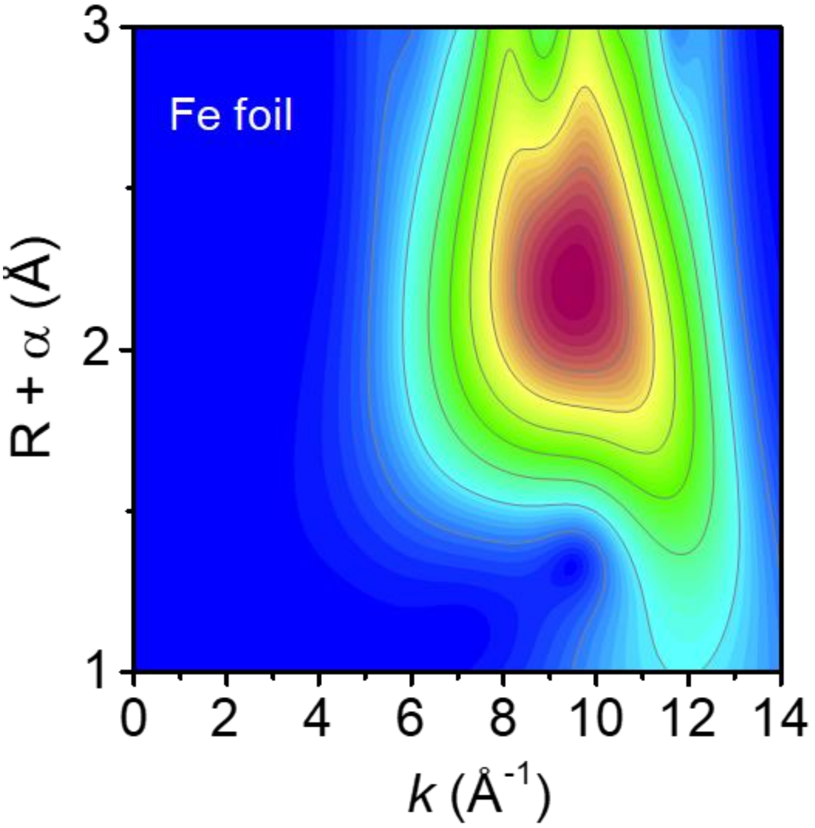

Supplementary Figure 10. k^3–weighted WT–EXAFS spectra of Fe foil at the Fe K–edge. The WT−EXAFS spectra of Fe foil showed the peak with a maximum intensity at 9.6 $Å^{-1}$, which is assigned to Fe−Fe scattering path.

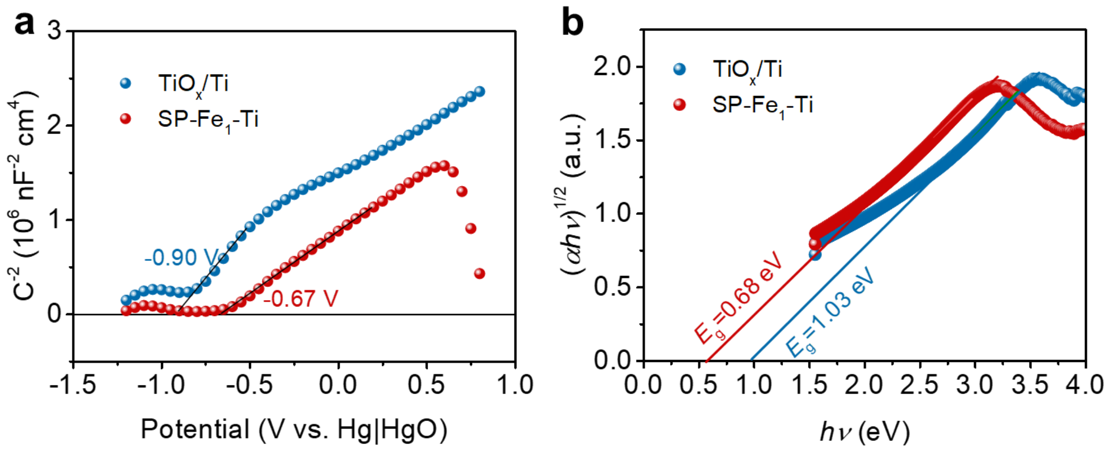

Supplementary Figure 11. The determination of band edge positions of TiO_x/Ti and SP–Fe_1–Ti electrodes. (a) Mott–Schottky plots of TiO_x/Ti and SP–Fe_1–Ti electrodes. CB positions of TiO_x/Ti and SP–Fe_1–Ti were determined through converting the flat–band potentials (vs. SHE) obtained from Mott–Schottky plots to CB positions (E_{CB}) that were usually ~0.1 eV higher than the flat–band potentials according to previous study[18]. The Fermi level (E_F) of N–type semiconductors is located close to conduction band minimum[18, 19]. (b) UV–vis–NIR spectra of TiO_x/Ti and SP–Fe_1–Ti electrodes. Bandgaps (E_g) of TiO_x/Ti and SP–Fe_1–Ti electrodes were calculated from the UV–vis–NIR spectra based on the Tauc equation[18]. VB positions (vs. SHE) of TiO_x/Ti and SP–Fe_1–Ti electrodes were calculated according to $E_{VB} = E_{CB} - E_g$. Finally, the band edge positions (vs. physical scale) of TiO_x/Ti and SP–Fe_1–Ti electrode were determined by the SHE (V)–physical scale (eV) transformation via $E_{phys} = -(E_{SHE} + 4.44)$[18].

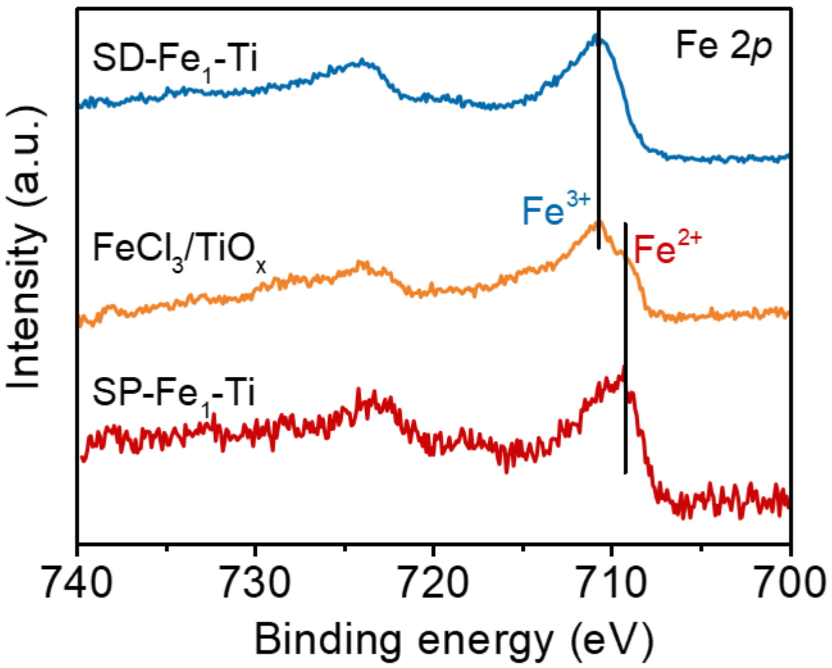

Supplementary Figure 12. Investigation of the interaction between oxygen vacancies and the loaded Fe ions. Fe 2p XPS spectra of $FeCl_3/TiO_x$ without thermal treatment, SD−Fe_1−Ti, and SP−Fe_1−Ti.

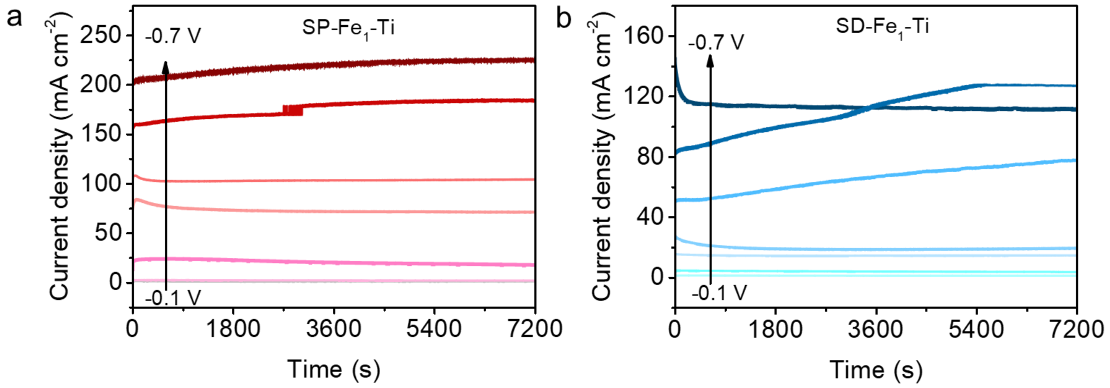

Supplementary Figure 13. The electrolysis curves at different applied potentials. The electrolysis curves of (a) SP−Fe_1−Ti and (b) SD−Fe_1−Ti at different applied potentials.

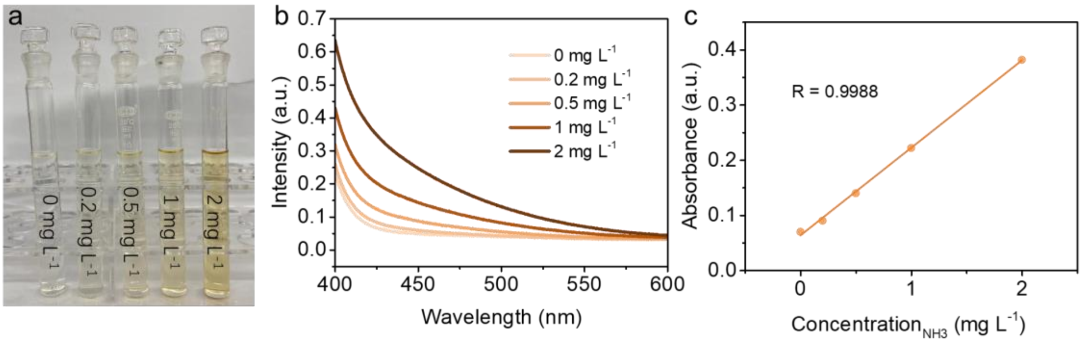

Supplementary Figure 14. Standard calibration curves for UV-Vis detection of NH_3. Standard calibration curves for UV-Vis detection of NH_3 from the Nessler's method (a) the standard solutions (b) raw UV-Vis spectra (c) linear calibration.

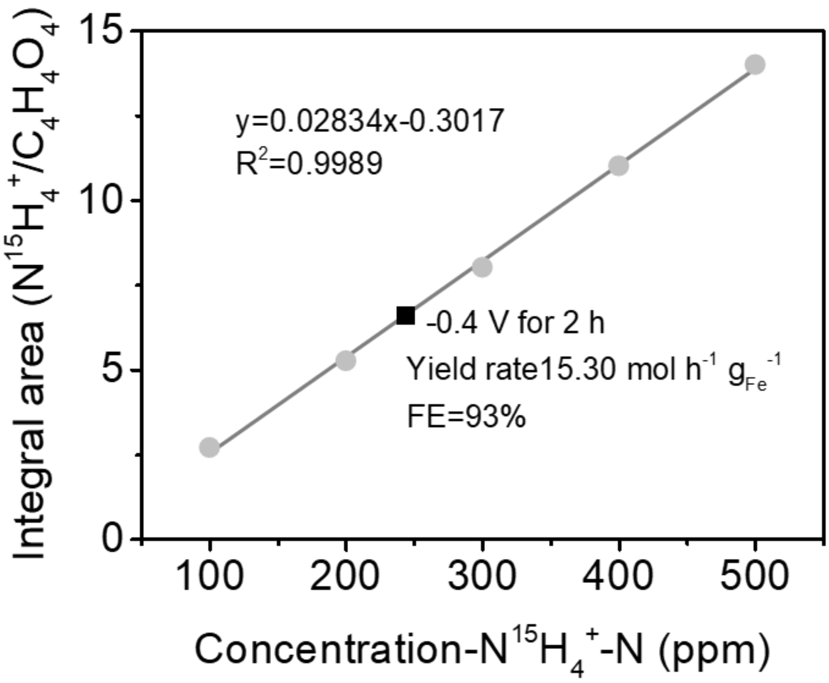

Supplementary Figure 15. The calibration curve used for estimation of NH_3 by $N^{15}H^{4+}$ ion of different concentrations. The maleic acid is selected as internal standard in 1H NMR test. With the assistance of ^{1}H nuclear magnetic resonance, SP−Fe_1−Ti electrode displayed an outstanding NH_3 yield rate of 15.3 mol_{NH3} g_{Fe}^{-1} h^{-1} and 93% faradaic efficiency at −0.4 V vs. RHE, which are in accordance with the results obtained from UV−Vis spectrophotometry.

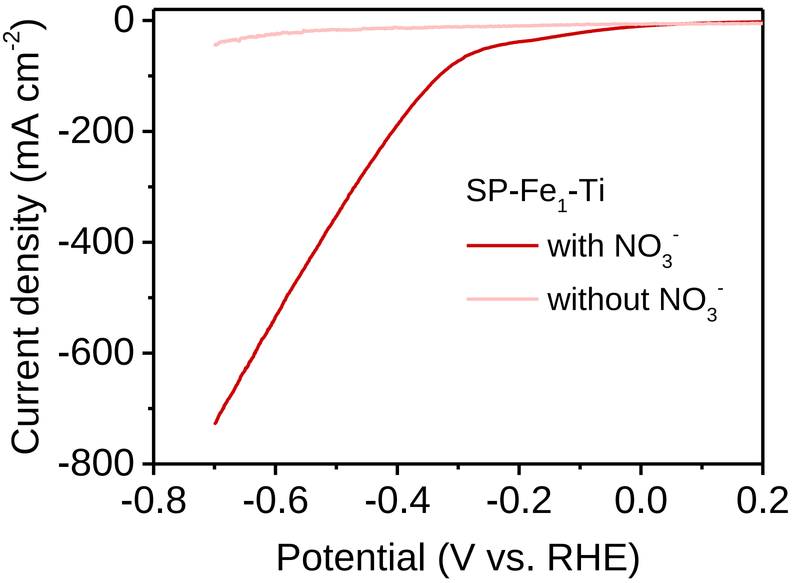

Supplementary Figure 16. LSV curves of SP−Fe1−Ti powders coated onto the carbon fiber paper. LSV curves of SP−Fe1−Ti powders coated onto the carbon fiber paper with and without addition of NO_3^-.

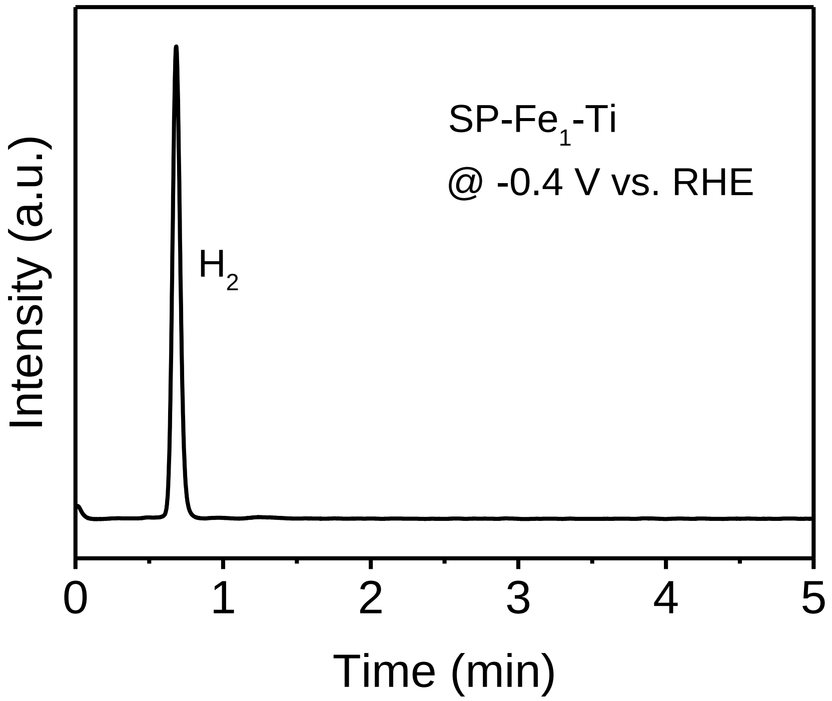

Supplementary Figure 17. The detection of gas products. Representative GC of gas products obtained on SP−Fe_1−Ti electrode at -0.4 V vs. RHE.

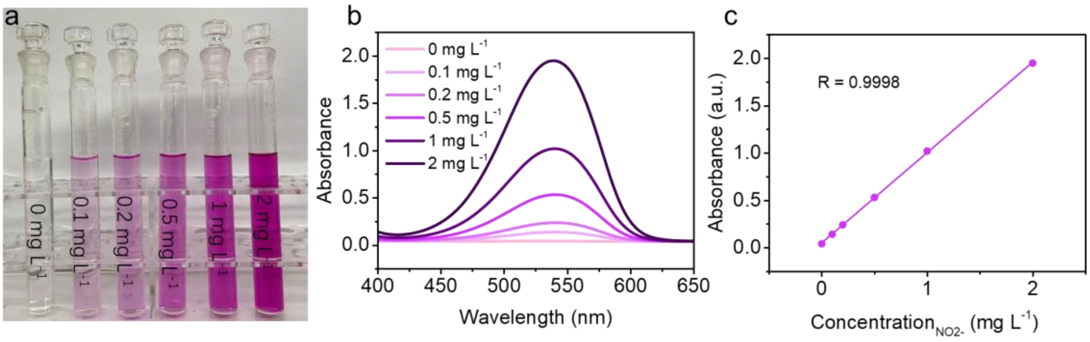

Supplementary Figure 18. Standard calibration curves for UV-Vis detection of NO_2^-. Standard calibration curves for UV-Vis detection of NO_2^- from the Griess's method (a) the standard solutions (b) raw UV-Vis spectra (c) linear calibration.

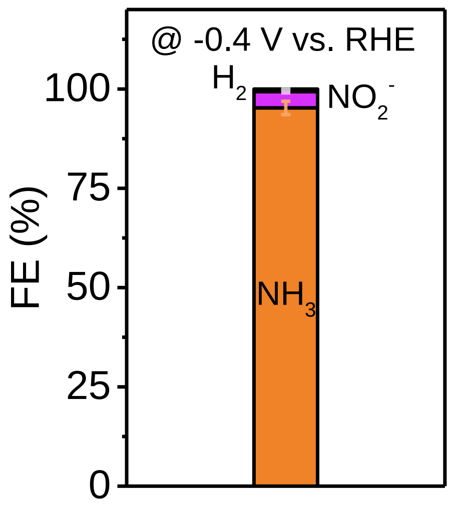

Supplementary Figure 19. The product distribution during NITRR on SP−Fe$_1$−Ti electrode. The FE of H_2 and NO_2^- and NH_3 during NITRR on on SP−Fe$_1$−Ti electrode at -0.4 V vs. RHE.

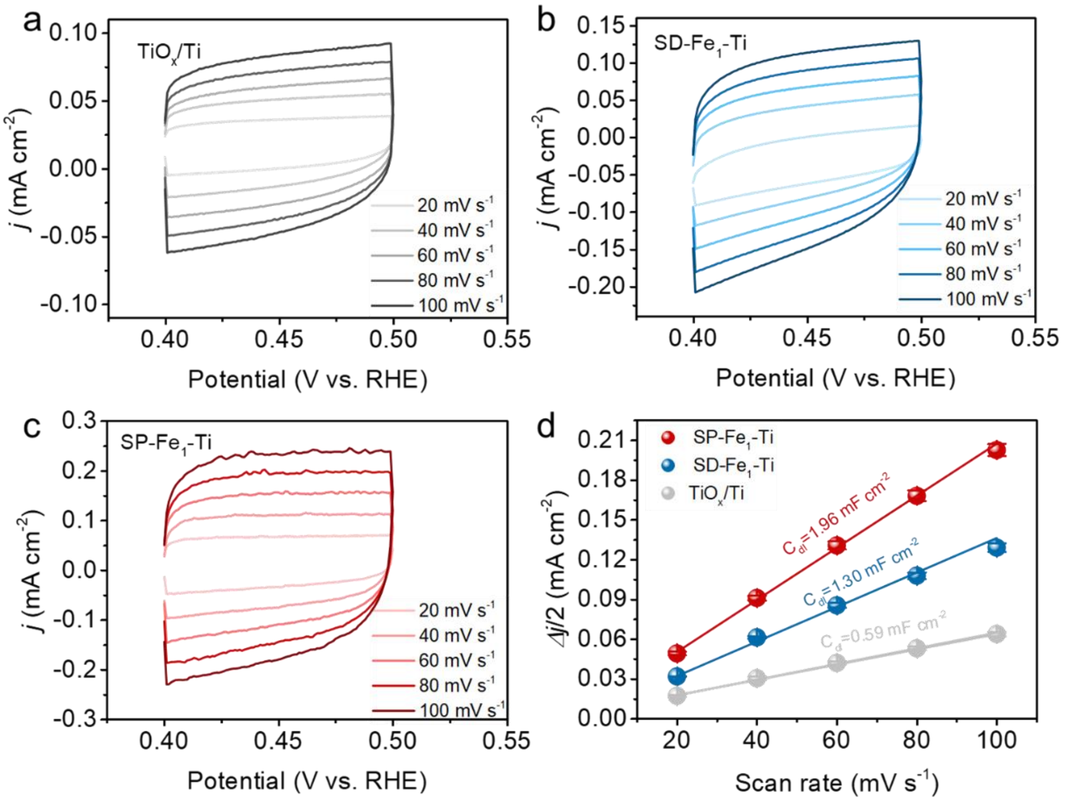

Supplementary Figure 20. Cyclic voltammetry curves at the scan rates changed from 20 to 100 mV s^{-1}. Cyclic voltammetry curves of (a) TiO_x/Ti, (b) and SD–Fe_1–Ti (c) SP–Fe_1–Ti electrodes. (d) Plots of half of the current density difference (Δj/2) at the centered potential plotted against the scan rate. The slope of fitted straight line is the C_{dl} value.

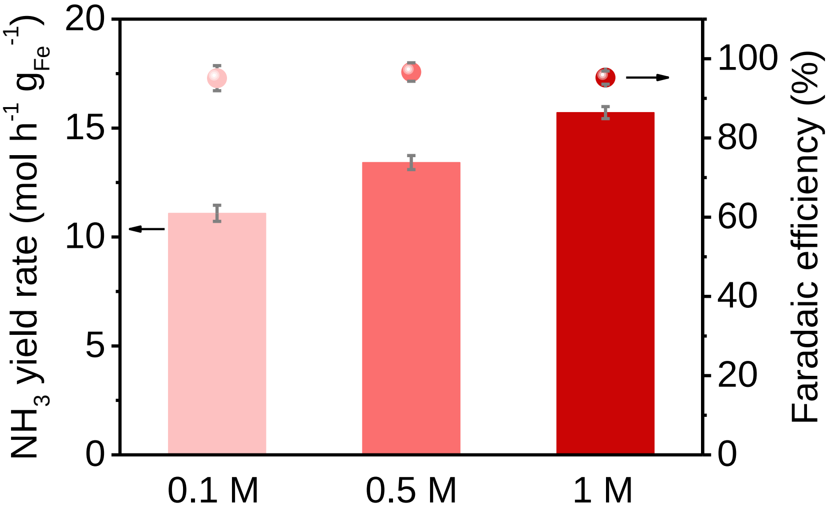

Supplementary Figure 21. The influence of nitrate concentration on electrode activity. NH_3 yield rate and FE_{NH3} of SP−Fe_1−Ti electrode at -0.4 V vs. RHE in 1 M KOH with addition of 0.1, 0.5, 1 M $NaNO_3$.

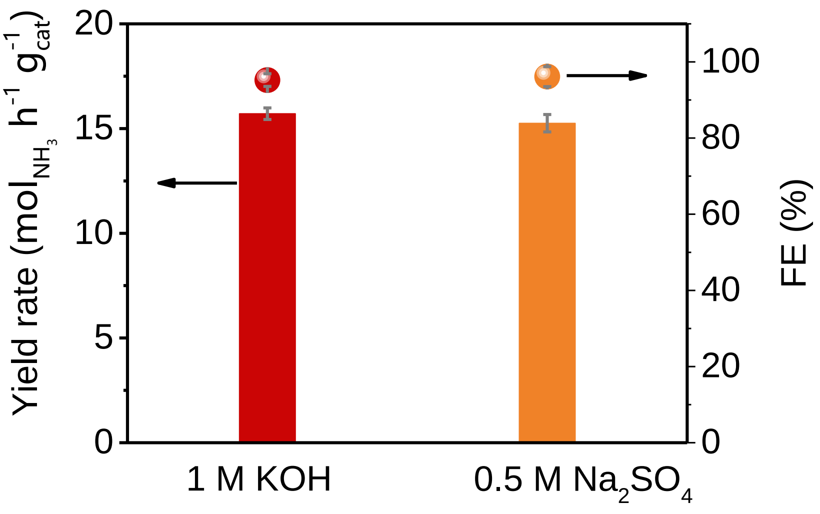

Supplementary Figure 22. The electrode activity under alkaline and neutral conditions. NH_3 yield rate and FE_{NH3} of SP−Fe_1−Ti electrode at -0.4 V vs. RHE in 1 M KOH and 0.5 M Na_2SO_4 with addition of 1 M $NaNO_3$.

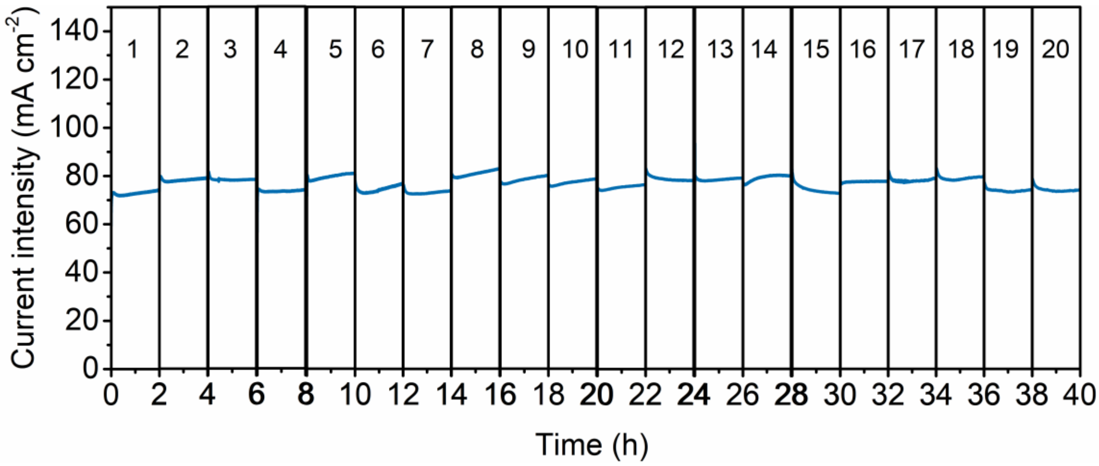

Supplementary Figure 23. Investigation of the electrode stability. The curves of cycling stability tests.

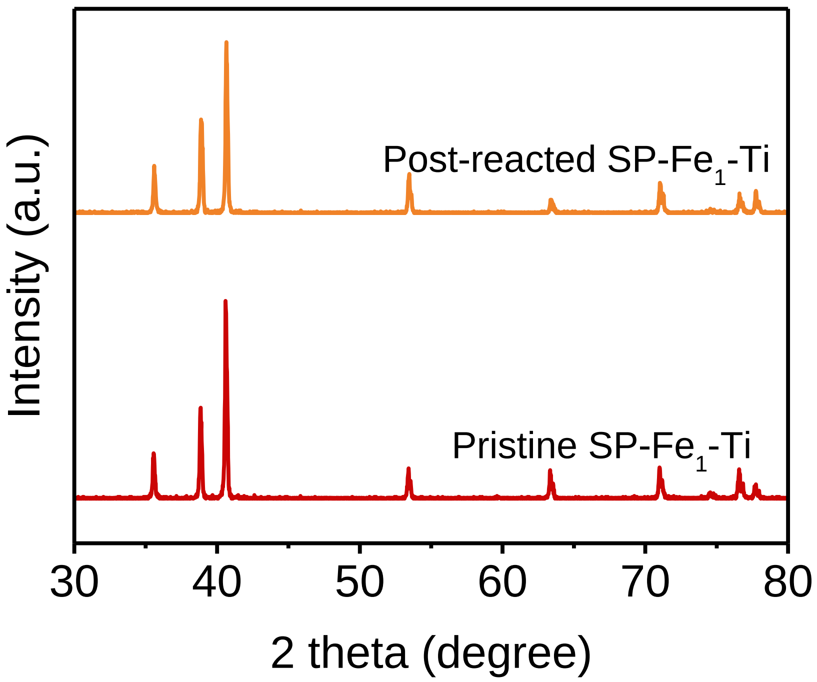

Supplementary Figure 24. Investigation of the electrode structure change after NITRR via XRD measurements. XRD patterns of pristine and post-reacted SP−Fe_1−Ti electrode.

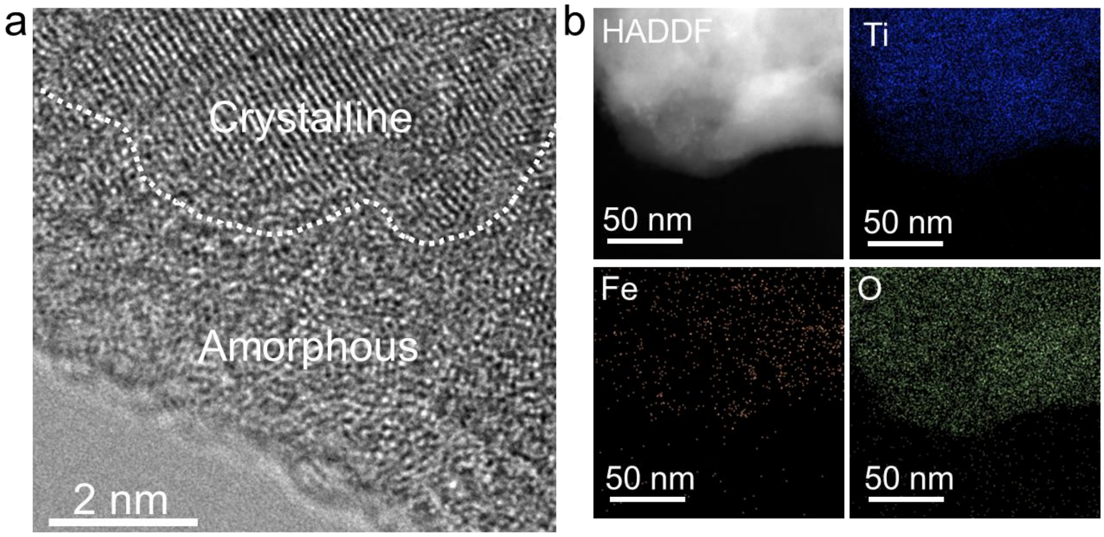

Supplementary Figure 25. Investigation of the electrode structure change after NITRR via TEM measurements. (a) HRTEM image and (b) HADDF-STEM image and corresponding elemental mapping of post-reacted SP−Fe_1−Ti electrode.

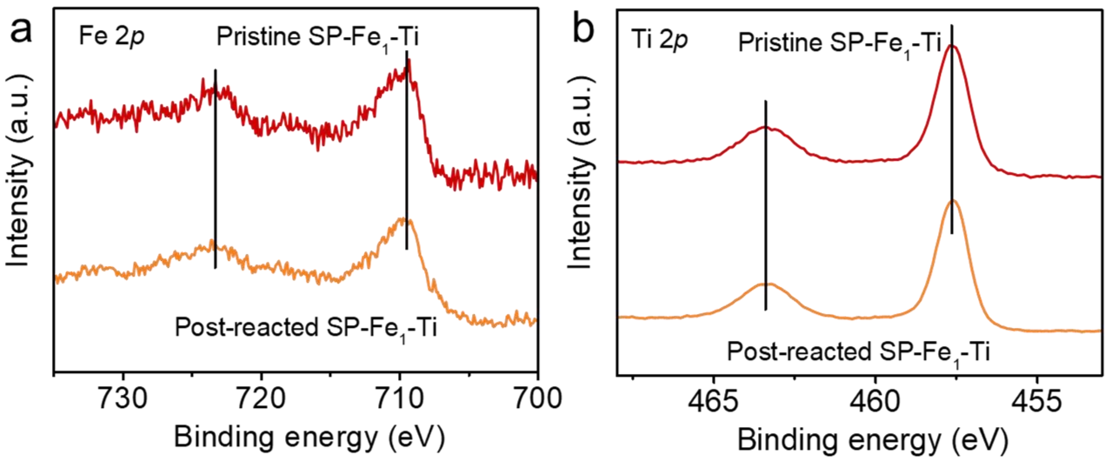

Supplementary Figure 26. Investigation of the electronic structure change of SP−Fe_1−Ti electrode after NITRR. (a) Fe 2p XPS spectra of pristine and post-reacted SP−Fe_1−Ti electrode. (b) Ti 2p XPS spectra of pristine and post-reacted SP−Fe_1−Ti electrode.

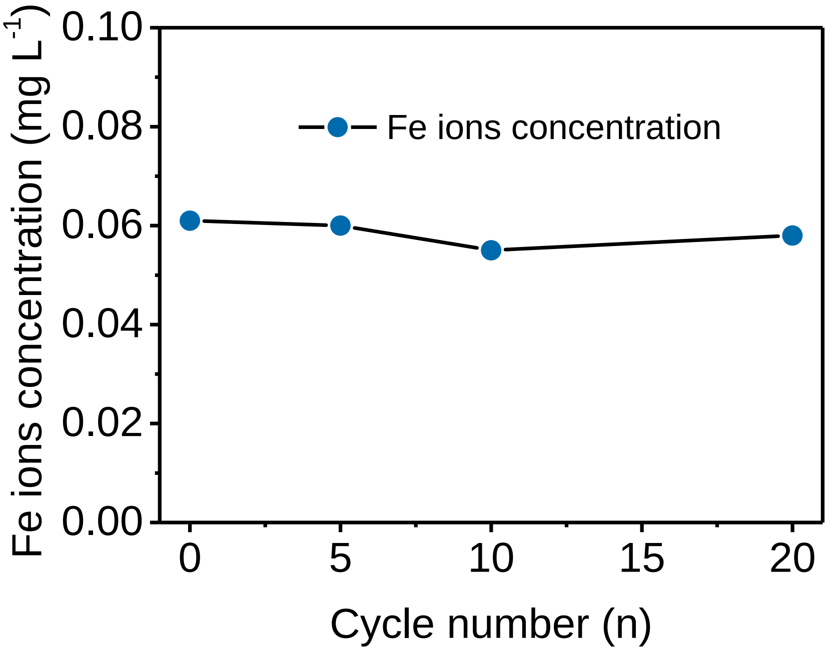

Supplementary Figure 27. Investigation of the Fe ions leaching during stability test. Fe ions concentrations of the pristine electrolyte and the post-reacted electrolyte after different cycles consecutive electrolysis.

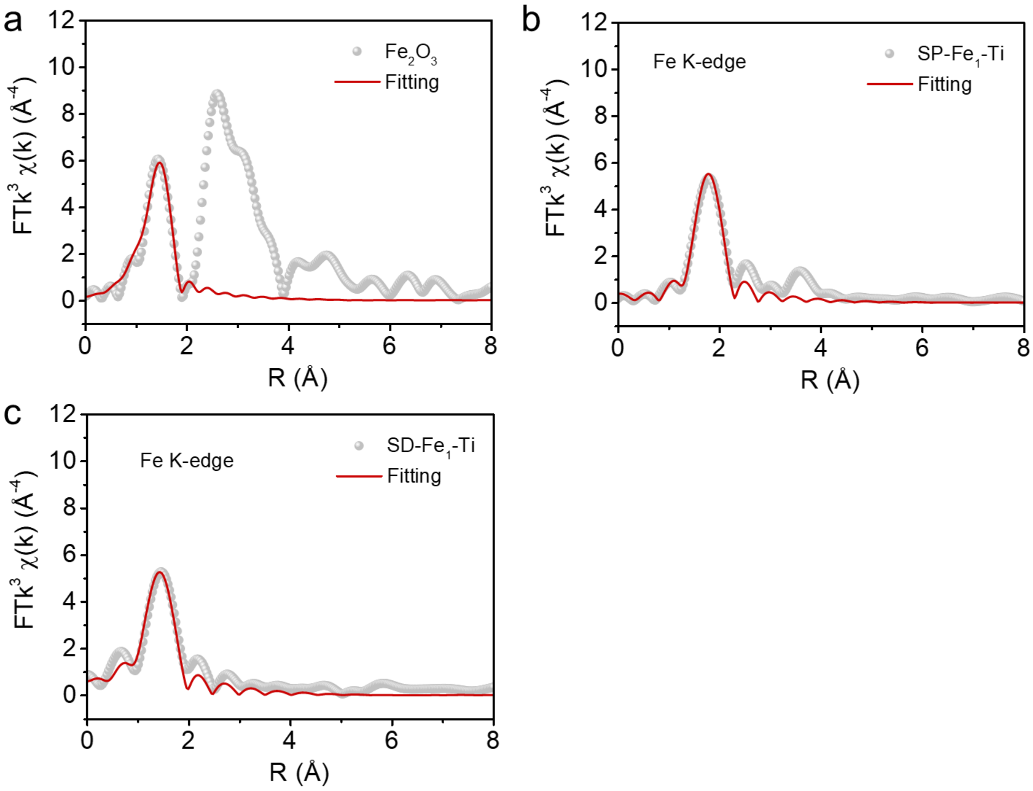

Supplementary Figure 28. The fitting results of EXAFS. R space fitting curves at Fe K–edge of (a) Fe_2O_3, (b) SP–Fe_1–Ti and (c) SD–Fe_1–Ti. Least square EXAFS fitting was performed to quantificational extract the local atomic structure parameters of SP–Fe_1–Ti and SD–Fe_1–Ti. It was found that the fitting curves matched quite well with the experiment spectra. The fitting structural parameters were displayed in Table S3.

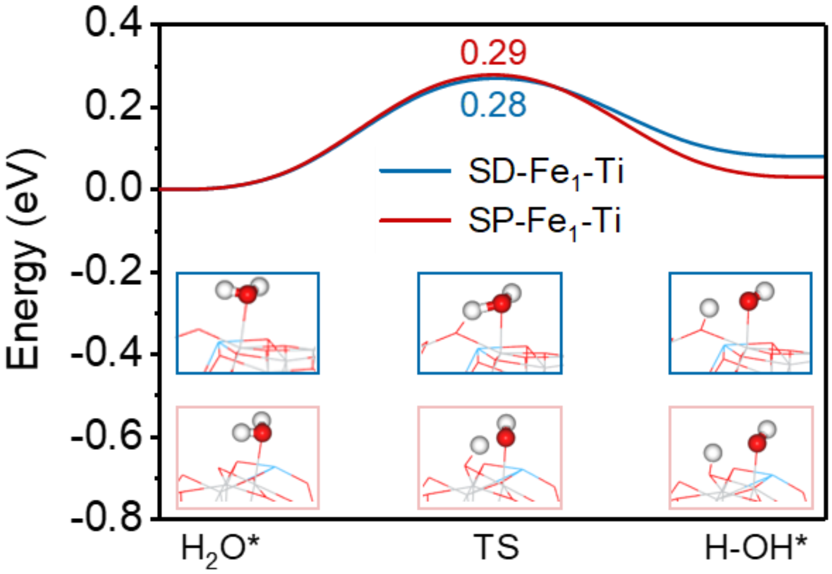

Supplementary Figure 29. Potential energy diagrams for H_2O dissociation on SD–Fe_1–Ti and SP–Fe_1–Ti. The insets are the optimized initial, transition and final states for H_2O dissociation. Color code: Ti (grey), Fe (cyan), N (blue), O (red) and H (white).

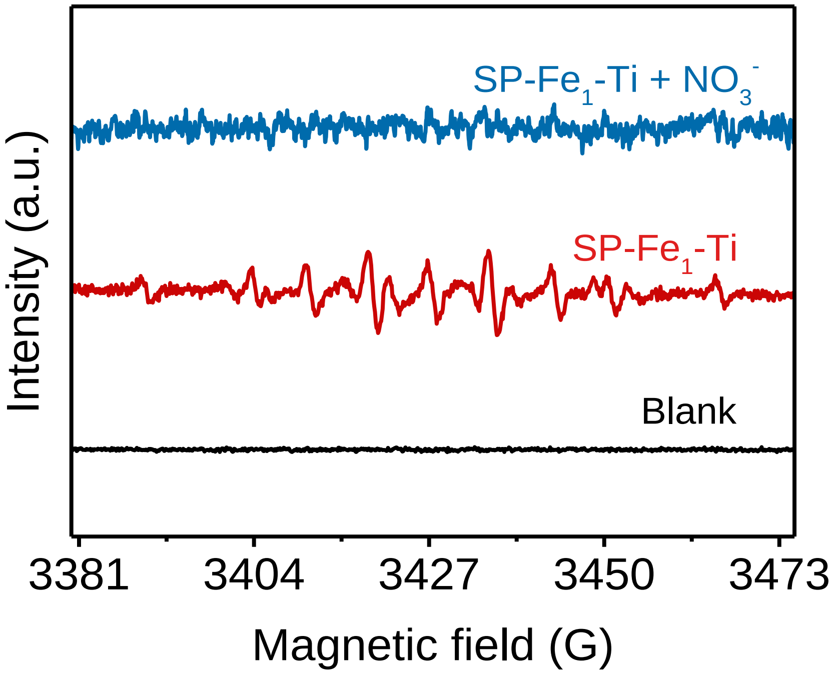

Supplementary Figure 30. Investigation of hydrogen radicals via ESR spectra. ESR spectra of pristine electrolyte, the electrolyte obtained after 10 min electrocatalysis on SP−Fe_1−Ti electrode in 1 M KOH without NO_3^- and the electrolyte obtained after 10 min electrocatalysis on SP−Fe_1−Ti electrode in 1 M KOH with NO_3^- under argon using DMPO as the ·H-trapping reagent.

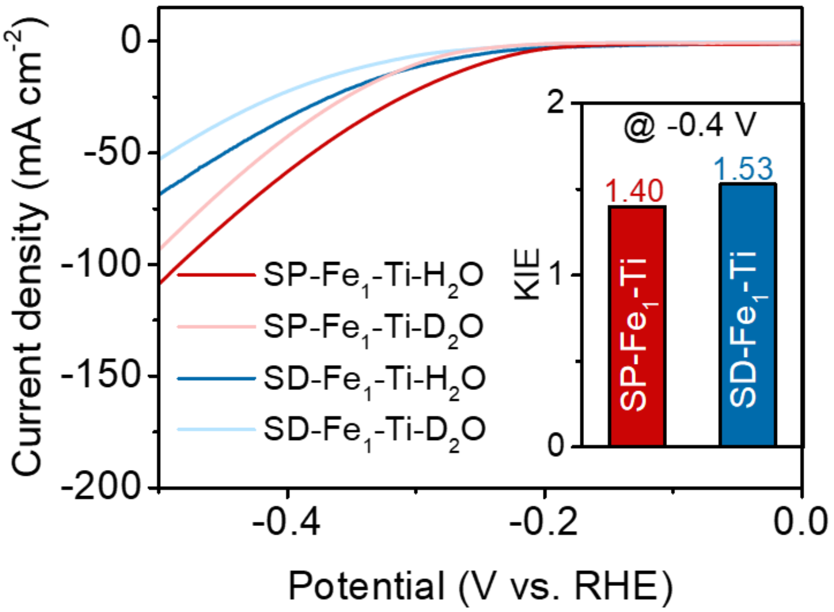

Supplementary Figure 31. Comparison of KIE values between SD–Fe$_1$–Ti and SP–Fe$_1$–Ti electrodes. LSV curves of SD–Fe$_1$–Ti and SP–Fe$_1$–Ti recorded in both H_2O and D_2O electrolytes with the addition of 1 mol L^{-1} $NaNO_3$ 1 mol L^{-1} KOH. Insert displays the KIE values obtained by calculating the current density ratios in H_2O and D_2O electrolytes of SD–Fe$_1$–Ti and SP–Fe$_1$–Ti electrodes at –0.4 V vs. RHE.

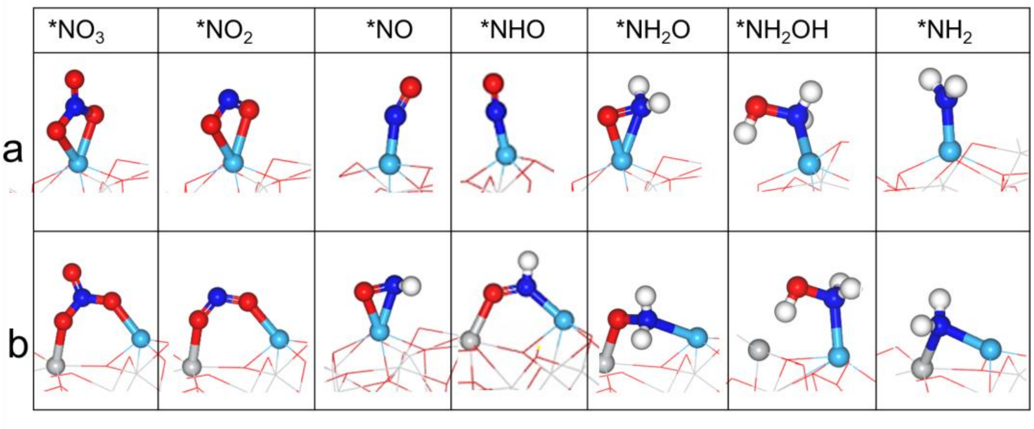

Supplementary Figure 32. The enlarged structures intermediates involving in NITRR. Optimized most stable structures for intermediates involving in NITRR on (a) SD–Fe_1–Ti and (b) SP–Fe_1–Ti. Color code: Ti (grey), Fe (cyan), N (blue), O (red) and H (white).

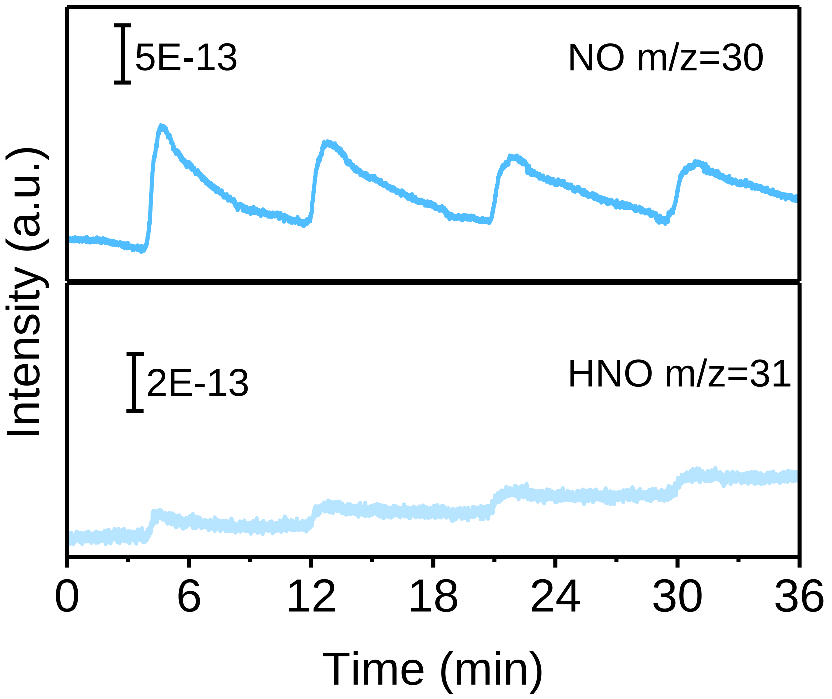

Supplementary Figure 33. The detection of key intermediates involving in NITRR via the DEMS measurement. DEMS signals of NO and HNO during NITRR over SD–Fe_1–Ti electrode.

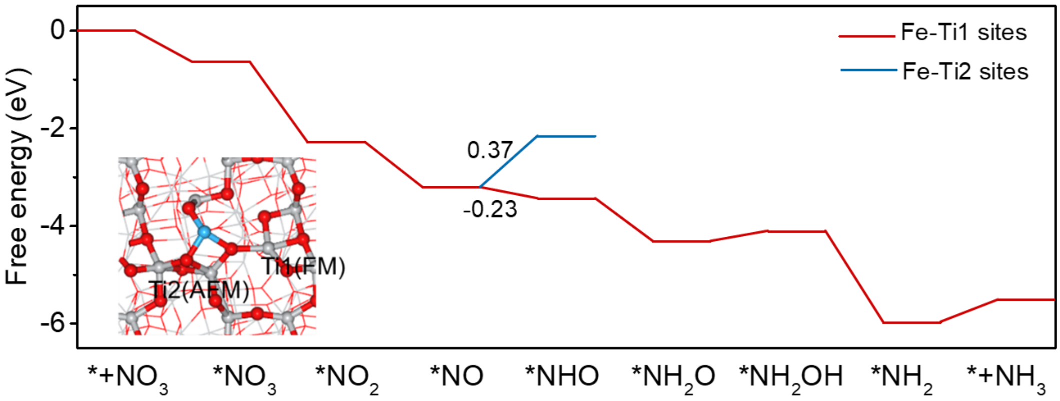

Supplementary Figure 34. Calculated free energy diagrams for NHO adsorbed on Fe–Ti1 and Fe–Ti2 pairs. Inset displayed the spin–polarized Ti1 and spin–depressed Ti2 atoms.

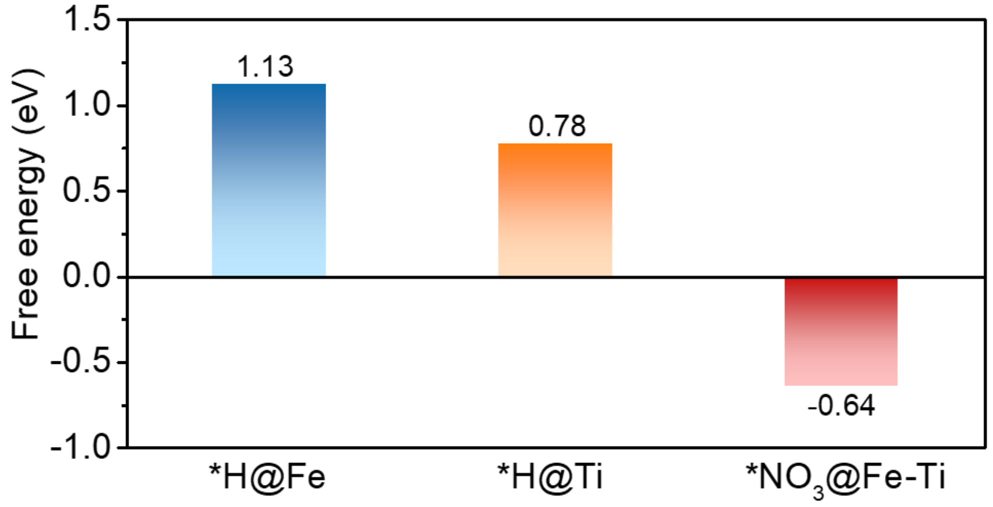

Supplementary Figure 35. HER vs. NITRR on SP–Fe_1–Ti pairs. The calculated free energies of *H on Fe and Ti sites of SP–Fe_1–Ti were 1.13 and 0.78 eV, respectively, much weaker than that of NO_3^- adsorbed on spin–polarized Fe–Ti pairs (–0.64 eV), suggesting its inhibiting effect on H_2 generation, and thus high FE can be obtained for NITRR on SP–Fe_1–Ti.

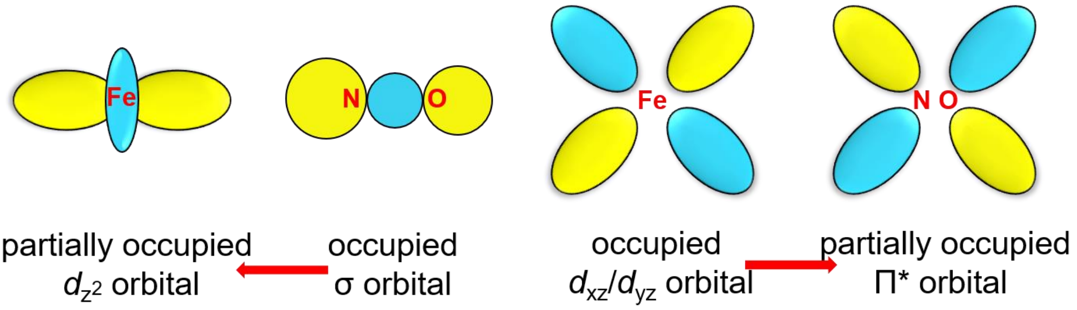

Supplementary Figure 36. The interaction between Fe and NO. Schematic mechanism of the interaction between Fe and NO.

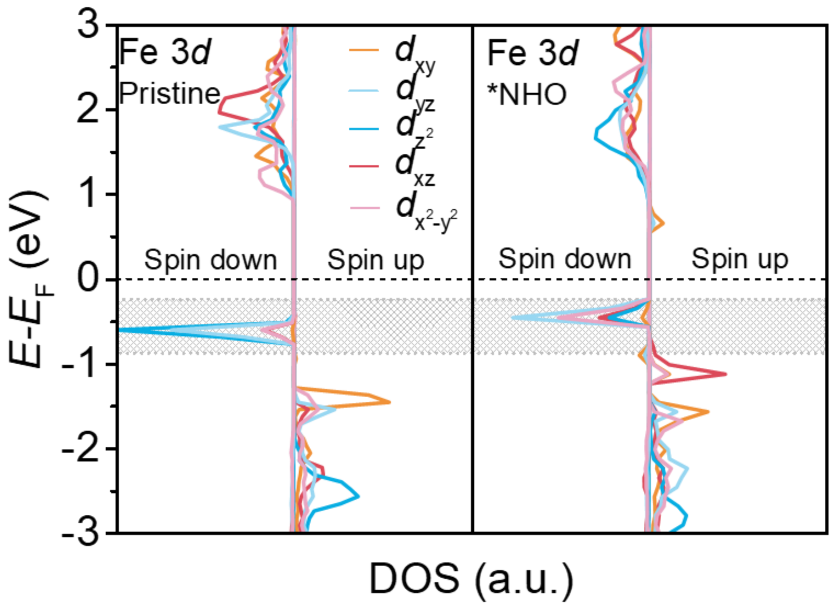

Supplementary Figure 37. DOSs change of Fe atom on SP−Fe_1−Ti after NHO adsorption. DOSs of Fe atom on SP−Fe_1−Ti before and after NHO adsorption.

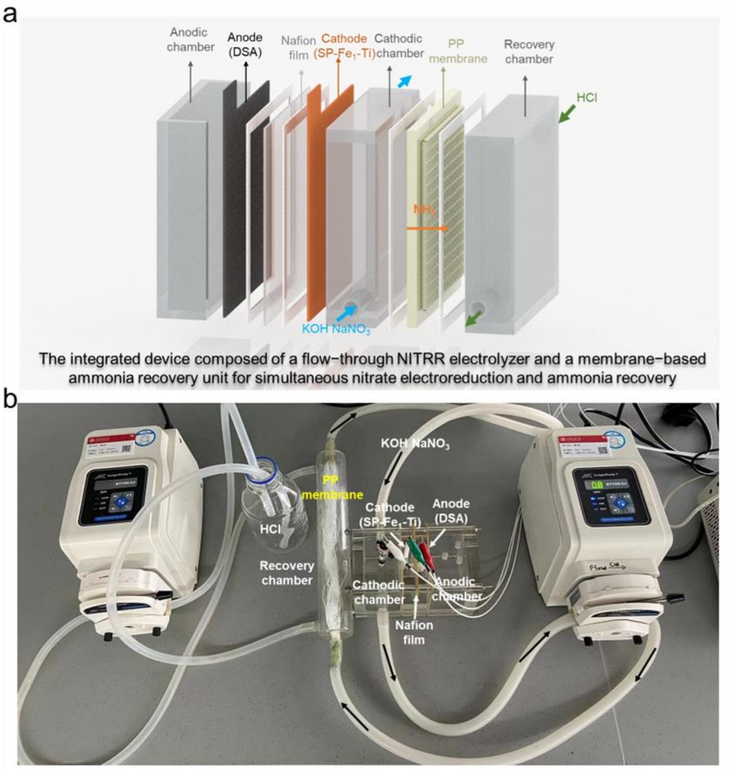

Supplementary Figure 38. The integrated device composed of a flow−through NITRR electrolyzer and a membrane−based ammonia recovery unit for simultaneous nitrate electroreduction and ammonia recovery. (a) A schematic diagram of the integrated device composed of a flow−through NITRR electrolyzer and a membrane−based ammonia recovery unit for simultaneous nitrate electroreduction and ammonia recovery. (b) Photograph of the integrated device for simultaneous nitrate electroreduction and ammonia recovery. The electrolyzer was an undivided cell of cathode and anode chambers (internal dimensions: 7 × 7 × 5 cm^3) made of plexiglass (poly (methyl methacrylate), PMMA). The SP-Fe_1-Ti cathode (2 × 2 cm^2) and the DSA anode (2 × 2 cm^2) were fixed into the chambers in a more compact manner (2-cm spacing) and separated by the nafion film (4.5 × 4.5 cm^2). The hollow polypropylene (PP) fibers were assembled into the home-made fiber arrays, which were placed in acidic solution to act as an NH_3 recovery reactor. The two open ends of the membrane arrays were fixed with epoxy resin and were connected with the electrolyzer by rubber tube.

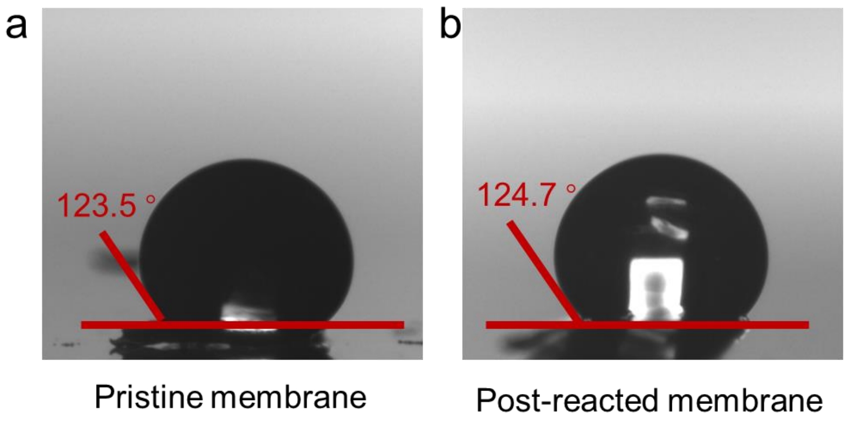

Supplementary Figure 39. The contact angle change after test. The contact angle of (a) the pristine and (b) post−reacted membrane.

Supplementary Tables

Supplementary Table 1. The electrical conductivity of electrode at room temperature.

Electrode	Electrical conductivity (kS cm^{-1})
SP−Fe_1−Ti	45
SD−Fe_1−Ti	43

Supplementary Table 2. Summary of the Mossbauer spectra parameters and assignments to different iron species on SD–Fe_1–Ti and SP–Fe_1–Ti electrodes.

Component		Assignment	IS (mm s^{-1})	QS (mm s^{-1})	Area (%)
SP–Fe_1–Ti	D1	HS Fe^{3+}	0.29	0.94	23.7
	D2	LS Fe^{3+}	0.038	0.959	36.1
	D3	HS Fe^{2+}	0.92	1.94	22.6
	D4	HS Fe^{2+}	0.846	1.28	17.6
SD–Fe_1–Ti	D1	HS Fe^{3+}	0.467	0.73	24.0
	D2	LS Fe^{3+}	0.13	0.72	50.0
	D3	HS Fe^{2+}	0.700	1.48	26.0

Supplementary Table 3. Comparison of the NITRR performance of SP–Fe_1–Ti electrode with the other NH_3 synthesis routes including Haber–Bosch process and nitrogen reduction reaction (NRR).

NH_3 synthesis routes	Catalysts	Electrolyte	FE_{NH3}	NH_3 yield rate (mmol gcat^{-1} h^{-1})	References
NITRR	SP–Fe_1–Ti	1 M KOH + 1 M $NaNO_3$	95.2% at –0.4 V vs. RHE	16000 mmol g_{cat}^{-1} h^{-1}at –0.4 V vs. RHE	This work
	Fe single atom	0.1 M K_2SO_4 + 0.5 M KNO_3	~ 75% at –0.66 V vs. RHE	308 mmol g_{cat}^{-1} h^{-1} at –0.66 V vs. RHE	*Nat. Commun.*, **2021**, 12, 2870.
	Fe–PPy SACs	0.1 M KOH + 0.1 M KNO_3	99.69% at –0.3 V vs. RHE	2507 mmol g_{cat}^{-1} h^{-1} –0.3 V vs. RHE	*Energy Environ. Sci.*, **2021**,14, 3522
	FeB	1 M KOH + 0.1 M KNO_3	96.8 % at –0.6 V vs. RHE	3150 mmol g_{cat}^{-1} h^{-1} at –0.6 V vs. RHE	*Angew. Chem. Int. Ed.*, **2023**, e202300054
	Fe_2TiO_5	PBS + 0.1 M $NaNO_3$	87.6 % at –0.9 V vs. RHE	730 mmol g_{cat}^{-1} h^{-1} at –1.0 V vs. RHE	*Angew. Chem. Int. Ed.*, **2023**, 62, e202215782
	SA–Fe(II)	0.1 M PBS + 0.5	99.6 % at –1.0 V	486 mmol g_{cat}^{-1} h^{-1} at –	*Proc. Natl. Acad. Sci.*,

	M Na_2SO_4 + 200 ppm $NaNO_3$	vs. RHE	1.0 V vs. RHE	**2023**, 120, e2209979120.
Co–Fe@Fe_2O_3	0.1 M Na_2SO_4 + 500 ppm $NaNO_3$	85.2% at –0.75 V vs. RHE	50 mmol g_{cat}^{-1} h^{-1} at –0.75 V vs. RHE	*Proc. Natl. Acad. Sci.*, **2022**, 119, e2115504119.
Cu–PTCDA	0.1 M PBS + 500 ppm KNO_3	77% at –0.4 V vs. RHE	130 mmol g_{cat}^{-1} h^{-1} at –0.4 V vs. RHE	*Nat. Energy.*, **2020**, 5, 605.
CuPd	1 M KOH + 1 M KNO_3	92.5% at –0.6 V vs. RHE	6250 mmol g_{cat}^{-1} h^{-1} at –0.6 V vs. RHE	*Nat. Commun.*, **2022**, 13, 2338.
CoP–CNS/ Cu foam	1 M NaOH + 1 M $NaNO_3$	88.6% at −1.03 V vs. RHE	3025 mmol g_{cat}^{-1} h^{-1} at −1.03 V vs. RHE	*Nat. Commun.*, **2022**, 13, 7958.
CoP NAs/CFC	1 M NaOH + 1 M $NaNO_3$	~100% at −0.3 V vs. RHE	569 mmol g_{cat}^{-1} h^{-1} at −0.3 V vs. RHE	*Energy Environ. Sci.*, **2022**,15, 760.
Ru_1–TiO_x/Ti	1 M KOH + 1 M $NaNO_3$	87.3% at –0.3V vs. RHE	22210 mmol g_{cat}^{-1} h^{-1} at –0.3V vs. RHE	*Angew. Chem. Int. Ed.* **2022**, *61*, e202208215
Strained Ru nanoclusters	1 M KOH + 1 M	~100% at –0.2 V	5560 mmol g_{cat}^{-1} h^{-1} at –	*J. Am. Chem. Soc.*, **2020**,

		KNO_3	vs. RHE	0.8 V vs. RHE	142, 7036.
NRR	Ru SACs on N doped carbon	0.05 M H_2SO_4	29.6% at –0.2V vs. RHE	7.1 mmol g_{cat}^{-1} h^{-1} at –0.2 V vs. RHE	*Adv. Mater.*, **2018**, 30, 1803498.
	Bi nanocrystals	0.5 M K_2SO_4	66% at –0.6 V vs. RHE	7.1 mmol g_{cat}^{-1} h^{-1} at –0.6 V vs. RHE	*Nat. Catal.*, **2019**, 2, 448
	$Bi_4V_2O_{11}/CeO_2$	0.1 M HCl	10.16% at –0.2V vs. RHE	1.4 mmol g_{cat}^{-1} h^{-1} at –0.6 V vs. RHE	*Angew. Chem. Int. Ed.*, **2018**, 57, 6073
Haber–Bosch process	Ru/Ba–$Ca(NH_2)_2$	/	/	60.7 mmol g_{cat}^{-1} h^{-1}	*Angew. Chem. Int. Ed.*, **2018**, 57, 2648.
	Co–LiH	/	/	4.7 mmol g_{cat}^{-1} h^{-1}	*Nat. Chem.*, **2017**, 9, 64

Supplementary Table 4. Comparison of the applied potentials for maximizing FE_{NH3}, onset potentials and current density of top−level NITRR electrocatalysts.

Catalysts	The applied potentials for maximizing FE_{NH3} (V vs. RHE)	The onset potential (V vs. RHE)	Current density @ -0.4 V vs. RHE (mA cm^{-2})	References
SP–Fe_1–Ti	–0.4	~–0.1	~75 (monolithic electrode) ~190 (powder)	This work
Fe single atom	–0.66	~–0.3	~5	Nat. Commun., 2021, 12, 2870.
Fe–PPy SACs	–0.3	~0.3	~12	Energy Environ. Sci., 2021,14, 3522.
FeB	–0.6	~–0.2	~150	Angew. Chem. Int. Ed., 2023, e202300054.
Fe_2TiO_5	–0.9	~–0.1	~18	Angew. Chem. Int. Ed., 2023, 62, e202215782
SA–Fe(II)	–1.0	~–0.3	~10	Proc. Natl. Acad. Sci., 2023, 120, e2209979120.
CuCl/TiO_2	–0.8	~–0.3	~8	Angew. Chem. Int. Ed. 2021, 60, 22933.

Cu/Cu_2O NWAs	–0.85	~–0.2	~35	Angew. Chem. Int. Ed.2020,59, 5350 – 5354
Cu–PTCDA	–0.4	0.27	~15	Nat. Energy., 2020, 5, 605.
Co–Fe@Fe_2O_3	–0.75	~–0.2	~5	Proc. Natl. Acad. Sci., 2022, 119, e2115504119.
CoP–CNS	−1.03	~0	~200	Nat. Commun., 2022, 13, 7958.
CoP NAs	−0.3	~0.1	~300	Energy Environ. Sci., 2022,15, 760.
Ru_1–TiO_x/Ti	–0.3	~0.1	~100	Angew. Chem. Int. Ed. 2022, 61, e202208215
Strained Ru	–0.2	~0.2	~110	J. Am. Chem. Soc., 2020, 142, 7036.
$Ru_{15}Co_{85}$ HNDs	0	~0.4	/	Nat Catal 2023, 6, 402.
CuCo nanosheet	–0.2 V	~0.1	/	Nat Commun 2022, 13, 7899
Ru-CuNW	0.04	~0.2	/	Nat. Nanotechnol. 2022, 17, 759.
Ru_1Cu_{10}/rGO	–0.05	~0.4	/	Adv. Mater. 2023, 35, 2202952.
CuPd	–0.6	~0	~180	Nat. Commun., 2022, 13, 2338.

Supplementary Table 5. Price of different metals[a]

Metal	Symbol	Unit of Measure	U.S.
Palladium	Pd	g	86.72 $
Ruthenium	Ru	g	24.11 $
Cobalt	Co	g	0.033 $
Copper	Cu	g	0.0082 $
Titanium	Ti	g	0.0063 $
Iron	Fe	g	0.00010 $

[a] The prices for various metals are from the metalary & tradingeconomics website on July 13, 2023. (https://www.metalary.com; https://tradingeconomics.com)

Supplementary Table 6. Comparison of the NH_3 yield rate defined by the electrode area among various electrode.

Catalysts	FE_{NH3}	NH_3 yield rate (mmol cm^{-2} h^{-1})	References
SP–Fe_1–Ti	98.51% at –0.4 V vs. RHE	0.99	This work
Fe single atom	~ 75% at –0.66 V vs. RHE	0.12	Nat. Commun., 2021, 12, 2870.
Fe–PPy SACs	99.69% at –0.3 V vs. RHE	0.16	Energy Environ. Sci., 2021,14, 3522
FeB	96.8 % at –0.6 V vs. RHE	1.5	Angew. Chem. Int. Ed., 2023, e202300054
Fe_2TiO_5	87.6 % at –0.9 V vs. RHE	0.073	Angew. Chem. Int. Ed., 2023, 62, e202215782
SA–Fe(II)	99.6 % at –1.0 V vs. RHE	0.29	Proc. Natl. Acad. Sci., 2023, 120, e2209979120.
CuCl/TiO_2	85 % at –0.8 V vs. RHE	0.13	Angew. Chem. Int. Ed. 2021, 60, 22933.
Cu/Cu_2O NWAs	95.8 % at –0.85 V vs. RHE	0.24	Angew. Chem. Int. Ed.2020,59, 5350 –5354
Cu–PTCDA	77 % at –0.4 V vs. RHE	0.026	Nat. Energy., 2020, 5, 605.
Co–Fe@Fe_2O_3	85.2 % at –0.75 V vs. RHE	0.089	Proc. Natl. Acad. Sci., 2022, 119, e2115504119.

CoP–CNS	88.6 % at −1.03 V vs. RHE	8.47	Nat. Commun., 2022, 13, 7958.
CoP NAs	~100 % at −0.3 V vs. RHE	3.09	Energy Environ. Sci., 2022,15, 760.
Ru_1–TiO_x/Ti	87.3 % at –0.3V vs. RHE	/	Angew. Chem. Int. Ed. 2022, 61, e202208215
Strained Ru	~100 % at –0.2 V vs. RHE	1.03	J. Am. Chem. Soc., 2020, 142, 7036.
$Ru_{15}Co_{85}$ HNDs	97 % at 0 V vs. RHE	1.92	Nat Catal 6, 402–414 (2023).
CuCo nanosheet	100 % at –0.2 V vs. RHE	4.8	Nat Commun 13, 7899 (2022)
Ru-CuNW	96 % at 0.04 V vs. RHE	4.5	Nat. Nanotechnol. 17, 759–767 (2022).
Ru_1Cu_{10}/rGO	98 % at –0.05 V vs. RHE	0.38	Adv. Mater. 2023, 35, 2202952.
CuPd	92.5 % at –0.6 V vs. RHE	1.25	Nat. Commun., 2022, 13, 2338.

Supplementary Table 7. Structural parameters extracted from the EXAFS fitting. (S_0^2=0.78 for Fe K–edge).

Samples	Scattering path	CN	R (Å)	$\sigma^2(10^{-3}Å^2)$	ΔE_0 (eV)	R factor
Fe_2O_3	Fe–O	6	1.95	9.8	–9.2	0.016
SP–Fe_1–Ti	Fe–O	3.3	1.95	3.1	–8.5	0.027
SD–Fe_1–Ti	Fe–O	4.1	1.93	5.1	–10.0	0.023

Notes: CN is the coordination number; R is interatomic distance; σ^2 is Debye–Waller factor (a measure of thermal and static disorder in absorber–scatterer distances); ΔE_0 is edge–energy shift (the difference between the zero kinetic energy value of the sample and that of the theoretical model); R factor is used to value the goodness of fitting.

Supplementary References

1. Yao Y., *et al.* Single atom Ru monolithic electrode for efficient chlorine evolution and nitrate reduction. *Angew. Chem. Int. Ed.* **61**, e202208215 (2022).
2. Kresse G., Furthmüller J. Efficiency of ab–initio total energy calculations for metals and semiconductors using a plane–wave basis set. *Comput. Mater. Sci.* **6**, 15–50 (1996).
3. Kresse G., Furthmüller J. Efficient iterative schemes for ab initio total–energy calculations using a plane–wave basis set. *Phys. Rev. B* **54**, 11169–11186 (1996).
4. Perdew J. P., Burke K., Ernzerhof M. Generalized gradient approximation made simple. *Phys. Rev. Lett.* **77**, 3865–3868 (1996).
5. Grimme S., Antony J., Ehrlich S., Krieg H. A consistent and accurate ab initio parametrization of density functional dispersion correction (DFT–D) for the 94 elements H–Pu. *J. Chem. Phys.* **132**, 154104 (2010).
6. Mavračić J., *et al.* Similarity between amorphous and crystalline phases: the case of TiO_2. *J. Phys. Chem. Lett.* **9**, 2985–2990 (2018).
7. Guo Z., Ambrosio F., Pasquarello A. Hole diffusion across leaky amorphous TiO_2 coating layers for catalytic water splitting at photoanodes. *J. Mater. Chem. A* **6**, 11804–11810 (2018).
8. Pham H. H., Wang L.–W. Oxygen vacancy and hole conduction in amorphous TiO_2. *Phys. Chem. Chem. Phys.* **17**, 541–550 (2015).
9. Prasai B., *et al.* Properties of amorphous and crystalline titanium dioxide from first principles. *J. Mater. Sci.* **47**, 7515–7521 (2012).
10. Dai J., *et al.* Single–phase perovskite oxide with super–exchange induced atomic–scale synergistic active centers enables ultrafast hydrogen evolution. *Nat. Commun.* **11**, 5657 (2020).
11. Dudarev S. L., *et al.* Electron–energy–loss spectra and the structural stability of nickel oxide: An LSDA+U study. *Phys. Rev. B* **57**, 1505–1509 (1998).
12. Wu T., *et al.* Greatly improving electrochemical N_2 reduction over TiO_2 nanoparticles by iron doping. *Angew. Chem. Int. Ed.* **58**, 18449–18453 (2019).
13. Nelson R., *et al.* LOBSTER: Local orbital projections, atomic charges, and chemical–bonding analysis from projector–augmented–wave–based density–functional theory. *J.*

Comput. Chem. **41**, 1931–1940 (2020).

14. Maintz S., Deringer V. L., Tchougréeff A. L., Dronskowski R. LOBSTER: A tool to extract chemical bonding from plane–wave based DFT. *J. Comput. Chem.* **37**, 1030–1035 (2016).
15. Guo S., *et al.* Insights into Nitrate reduction over indium–decorated palladium nanoparticle catalysts. *ACS Catal.* **8**, 503–515 (2018).
16. Liu J.–X., Richards D., Singh N., Goldsmith B. R. Activity and selectivity trends in electrocatalytic nitrate reduction on transition metals. *ACS Catal.* **9**, 7052–7064 (2019).
17. Niu H., *et al.* Theoretical insights into the mechanism of selective nitrate–to–ammonia electroreduction on single–atom catalysts. *Adv. Funct. Mater.* **31**, 2008533 (2021).
18. Mao C., *et al.* Energy–confined solar thermal ammonia synthesis with K/Ru/$TiO_{2-x}H_x$. *Appl. Catal. B Environ.* **224**, 612–620 (2018).
19. Persson C., Zhao Y.–J., Lany S., Zunger A. N–type doping of $CuInSe_2$ and $CuGaSe_2$. *Phys. Rev. B* **72**, 035211 (2005).
